# Pipsqueak family genes *dan/danr* antagonize nuclear Pros to prevent neural stem cell aging in *Drosophila* larval brains

**DOI:** 10.3389/fnmol.2023.1160222

**Published:** 2023-05-17

**Authors:** Huanping An, Yue Yu, Xuming Ren, Minghua Zeng, Yu Bai, Tao Liu, Huimei Zheng, Rong Sang, Fan Zhang, Yu Cai, Yongmei Xi

**Affiliations:** ^1^Key Laboratory of Clinical Molecular Biology of Hanzhong City, Hanzhong Vocational and Technical College, Hanzhong, China; ^2^Department of Teaching and Medical Administration, 3201 Hospital, Xi’an Jiaotong University Health Science Center, Hanzhong, China; ^3^School of Biological Science and Engineering, Shaanxi University of Technology, Hanzhong, China; ^4^The Women’s Hospital, Institutes of Genetics, School of Medicine Zhejiang University, Hangzhou, China; ^5^Temasek Life Sciences Laboratory, National University of Singapore, Singapore, Singapore

**Keywords:** *Drosophila*, neural stem cells, premature aging, *dan/danr*, Prospero

## Abstract

Neural stem cell aging is a fundamental question in neurogenesis. Premature nuclear Pros is considered as an indicator of early neural stem cell aging in *Drosophila*. The underlying mechanism of how neural stem cells prevent premature nuclear Pros remains largely unknown. Here we identified that two pipsqueak family genes, *distal antenna* (*dan*) and *distal antenna-related* (*danr*), promote the proliferation of neural stem cells (also called neuroblasts, NBs) in third instar larval brains. In the absence of Dan and Danr (*dan/danr*), the NBs produce fewer daughter cells with smaller lineage sizes. The larval brain NBs in *dan/danr* clones show premature accumulation of nuclear Prospero (Pros), which usually appears in the terminating NBs at early pupal stage. The premature nuclear Pros leads to NBs cell cycle defects and NB identities loss. Removal of Pros from *dan/danr* MARCM clones prevents lineage size shrinkage and rescues the loss of NB markers. We propose that the timing of nuclear Pros is after the downregulation of *dan/danr* in the *wt* terminating NBs. *dan/danr* and nuclear Pros are mutually exclusive in NBs. In addition, *dan/danr* are also required for the late temporal regulator, Grainyhead (Grh), in third instar larval brains. Our study uncovers the novel function of *dan/danr* in NBs cell fate maintenance. *dan/danr* antagonize nuclear Pros to prevent NBs aging in *Drosophila* larval brains.

## 1. Introduction

The precisely regulated proliferative status of neural stem cells plays a pivotal role in neurogenesis. Any disturbances of neural stem cell fate will lead to progeny number defects (Holguera and Desplan, 2018; Dray et al., 2021). *Drosophila* neural stem cells, termed neuroblasts (NBs), provide a unique model system to study the mechanisms involved in neural stem cell maintenance (Homem et al., 2014; Wu et al., 2019; Liu et al., 2020; Maurange, 2020; Sang et al., 2022). Nuclear Pros has been recognized as an indicator of the end of the NB lifespans (Li and Vaessin, 2000; Cenci and Gould, 2005; Maurange et al., 2008; Chai et al., 2013; Wu et al., 2019; Sang et al., 2022). During NB asymmetric divisions, Pros is always cytoplasmic and then is segregated exclusively into ganglion mother cells (GMCs) after each round of cell division (Hirata et al., 1995; Spana and Doe, 1995; Ikeshima-Kataoka et al., 1997). Whenever nuclear Pros is observed in NBs, the consensus is that these cells will soon undergo terminal symmetric division and terminate their NB cell fate (Li and Vaessin, 2000; Lai and Doe, 2014). Thus, one of the important roles for the maintenance of NB proliferation is to prevent the premature accumulation of Pros in the nuclei of NBs. The regulatory mechanism of how NBs prevent premature nuclear Pros remains largely unknown.

In central brains of *Drosophila* larvae there are two types of NBs: type I and type II (Bello et al., 2008; Boone and Doe, 2008; Doe, 2008; Izergina et al., 2009). Type I NBs represent the majority of neural stem cells (~90/lobe) and exhibit specific markers such as Asense (Ase) and Deadpan (Dpn). Type II NBs have a smaller population (8/lobe) and express Dpn, but not Ase (Boone and Doe, 2008). All of these NBs undergo proliferation at larval stages and terminate their respective stem cell fates at the early pupal stage (Maurange et al., 2008; Chai et al., 2013; Wu et al., 2019). We have previously reported that RanGAP, a nucleocytoplasmic transport regulator, is involved in Pros accumulation in the nuclei, and knockdown of RanGAP resulted in premature nuclear Pros and a short lifespan of NBs (Wu et al., 2019).

The cell fate of NBs is precisely maintained along the developmental axis together with temporal regulation (patterning), as a cascade of transcription factors that are sequentially expressed in NBs that specify NBs identities (Isshiki et al., 2001), and proliferative status (Maurange et al., 2008; Bakshi et al., 2020). Temporal regulation factors are divided into two groups. The first group promotes and ensures the scheduled NB termination in the early pupal brain. This prevents an undesired prolonged lifespan for NBs as occurs, for example, in the absence of Cas or Svp, when temporal scheduling is halted and prolonged NB lifespans result (Maurange et al., 2008). Conversely, the second group acts to safeguard NB cell fate and avoids early NB termination. Lack of these latter factors leads to the premature NB decommission and results in a shortened NB lifespan. Grh is an example of latter group (Cenci and Gould, 2005; Bakshi et al., 2020).

The pipsqueak domain family genes contain DNA binding motifs and transcriptionally regulate genes expression, that are conserved between invertebrate and vertebrate species (Siegmund and Lehmann, 2002). In *Drosophila*, two pipsqueak domain gene family members *dan/danr* share a large proportion of protein sequences and exhibit redundant functions (Siegmund and Lehmann, 2002; Emerald et al., 2003; Suzanne et al., 2003; Kohwi et al., 2011). During the *Drosophila* embryonic neurogenesis, *dan/danr* regulate Hunchback (Hb) expression (Kohwi et al., 2011). *dan/danr* are also known to be involved in transcription regulation networks (Emerald et al., 2003; Suzanne et al., 2003).

In this study, we identified that *dan/danr* play important roles in NBs fate maintenance in *Drosophila* third instar larval brains. *dan/danr* act to prevent early accumulation of Pros in NB nuclei. Lack of *dan/danr* causes premature nuclear Pros accumulation in NBs which results in defective NB cell cycles and loss of the NB markers Ase and Dpn. *dan/danr* MARCM clones showed that removal of Pros is able to prevent NB marker loss and small lineage phenotypes. *dan/danr* antagonizes the function of nuclear Pros to maintain NB cell fates in third instar larval brains.

## 2. Materials and methods

### 2.1. *Drosophila* strains

All fly stocks and crosses were maintained at 25°C. We used the following *Drosophila* strains to analyze the phenotypes: *danr^ex35^*, *dan/danr^ex56^*, and *dan^emS3^* (Emerald et al., 2003). We recombined *dan/danr^ex56^* together with *FRT* 82B to conduct MARCM analysis in the larval brains. Other mutant lines including: *grh^1M^ FRT* 42D / Cyo GFP, *cas^24^ FRT* 82B/TM6B, *svp^1^ FRT* 82B/TM6B (Chai et al., 2013) and *pros^17^ FRT* 82B / TM6B (all shared by Dr. Cai) were used to analyze the NB clone phenotypes. The following Gal4 lines used to analyze the gene expression patterns or to overexpress the genes in NBs: *ase*-Gal4, *wor*-Gal4 (Bloomington stock center). The *UAS*-CD8::GFP line (THJ0080) was used to label the cells. The balancer lines used in this paper were TM3*^sb^*/TM6B and Gla/Cyo (Bloomington stock center). *FRT* 42D and *FRT* 82B were used to induce clones in larval brains as controls.

### 2.2. Clonal analysis

MARCM clones were generated according to the standard method (Lee and Luo, 2001). Once the cage had been set up, *Drosophila* embryos were collected over a period about 6–8 h and then the embryos were kept at room temperature (RT). Heat-shock was conducted at around 24–30 h after larval hatching (ALH) at 37°C in water bath for between 30 min to 1 h as indicated. Dissection of the larval brains was conducted at the indicated time points (72 h, 96 h or 120 h ALH) based on the corresponding genotypes, and then fixed for immunostaining.

### 2.3. Immunofluorescence staining and imaging

Brains were fixed 15 min at 4% paraformaldehyde in 0.1 M HEPES pH 7.4. Antibody staining was performed according to the reported methods (An et al., 2017). The primary antibodies, dilutions, and sources used in this assay were: rabbit anti-Dan/Danr 1/1000; guinea-pig anti-Dan/Danr 1/1000; mouse anti-Pros 1/10; mouse anti-Elav (44C11) 1/10; rabbit anti-Caspase-3 1/1000 (Abcam); guinea anti-Dpn 1/1000 (Y. Cai’s lab); rabbit anti-Ase 1/1000; rabbit anti-Grh 1/1000 (Y. Cai’s lab); mouse, rabbit and Chicken anti-GFP (Abcam) and rabbit anti-phospho-histone H3 (PH3) (Abcam). Secondary antibodies were conjugated to either Alexa Fluor 488, Alexa Fluor 555, or Alexa Fluor 633 (Molecular Probes), and used at 1/500, 1/1,000, or 1/250, respectively. TO-PRO-3 (Molecular Probes) at 1/5,000 was used for DNA staining and samples were mounted in Vectashield (Vector Laboratories). Images were obtained using OLMPUS upright microscope (FV-1000) and processed in Adobe Photoshop 2021. EdU incorporation was performed as per the kit instructions (Invitrogen).

### 2.4. Quantification and statistical analysis

Statistical analysis was performed in Graphpad Prism9. Data are presented as the mean ± SEM. Unpaired Student’s *t*-test and ordinary one-way ANOVA or 2way ANOVA test were performed to assess differences. A *p* value of <0.05 was considered statistically significant. Three replicates were conducted in each statistic group. All of the statistical details of experiments can be found in the figure panels and legends.

## 3. Results

### 3.1. *dan/danr* are required for NB proliferation

**Figure 1:**
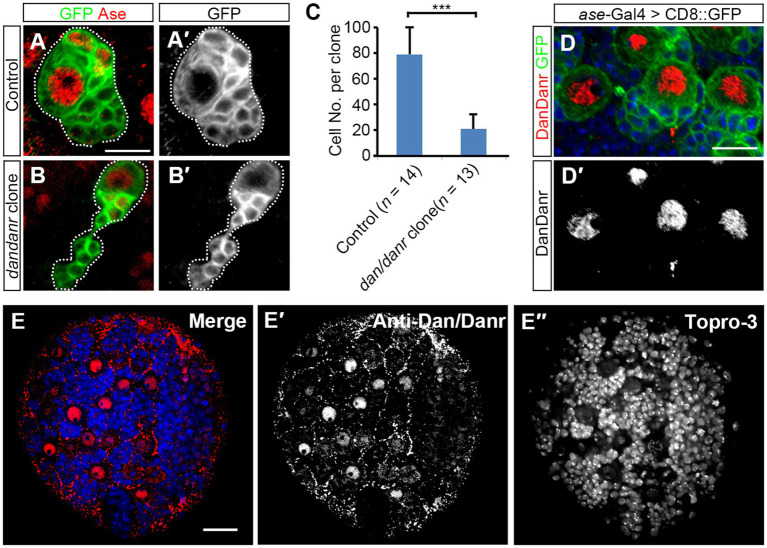
NB proliferation defects are found in *dan/danr* NBs. **(A,B′)** MARCM clones of type I NB labeled by GFP (green) in *Drosophila* third instar larval brains. Compared with controls **(A,A′)**, the size of *dan/danr* MARCM clone **(B,B′)** is smaller. NBs with Ase staining (red) are type I NBs. Dotted lines outline MARCM clones. **(C)** The statistical data of the average total cell number within a single lineage between control (*n* = 14) and *dan/danr* (*n* = 13) clones. Data are presented as mean ± SEM. Unpaired student t-test was performed (***p value <0.001). Only about a quarter of cells remains in *dan/danr* clones as compared with the controls. **(D,D′)** Double labeling of anti-Dan/Danr (red) and GFP (green) in third instar larval brain NBs. GFP driven by *ase*-Gal4 marks type I NB lineages. Dan/Danr are expressed in type I NBs. Scale bars: 10 μm. **(E–E″)** Anti-Dan/Danr immunofluorescence staining pattern in larval brain. Dan/Danr were expressed in larval brain NBs. Scale bar: 20 μm. Topro-3 (blue) labels DNA.

*dan* and *danr* are *Drosophila* pipsqueak domain family genes and share highly conserved protein sequences (Emerald et al., 2003). According to FlyAtlas2 anatomy RNA-Seq data^1^ and the previous report (Emerald et al., 2003), *dan* and *danr* are mainly expressed in the larval central nervous system (CNS). To explore the function of *dan/danr* in CNS development in larval brains, we generated *dan/danr* MARCM clones in NBs. Since type I NBs represent the majority of NBs in the central brain, we only focused on type I NB clones. We found the mutant NB lineages to be smaller in size than those of controls (Figures 1A,B). In addition, on average each *dan/danr* clone contained only 20 ± 7 (*n* = 13) cells whereas approximately 85 ± 13 (*n* = 14) cells were found in the controls at 96 h ALH (Figure 1C).

We then generated the antibody and performed immunostaining to show the dynamic expression pattern of Dan/Danr. The fusion protein containing the full length of the amino acid sequence of Dan was used as the antigen to raise the antibodies. The antibodies recognized both Dan and Danr proteins due to their sequence similarities (Supplementary Figures S1A–D). Anti-Dan/Danr staining showed that they are highly expressed in NBs of the third instar larval brains (Figures 1D,E).

In order to test whether the smaller sized *dan/danr* clones are due to cell apoptosis, we performed Caspase-3 immunofluorescence staining (Supplementary Figure S2). Results showed that there was no difference in Caspase-3 signals between the *dan/danr* clones and the control clones, indicating that no apoptosis has occurred. These data suggest the absence of *dan/danr* affected NB proliferation and led to smaller NB clone size.

### 3.2. Lack of *dan/danr* leads to nuclear pros in NBs

**Figure 2:**
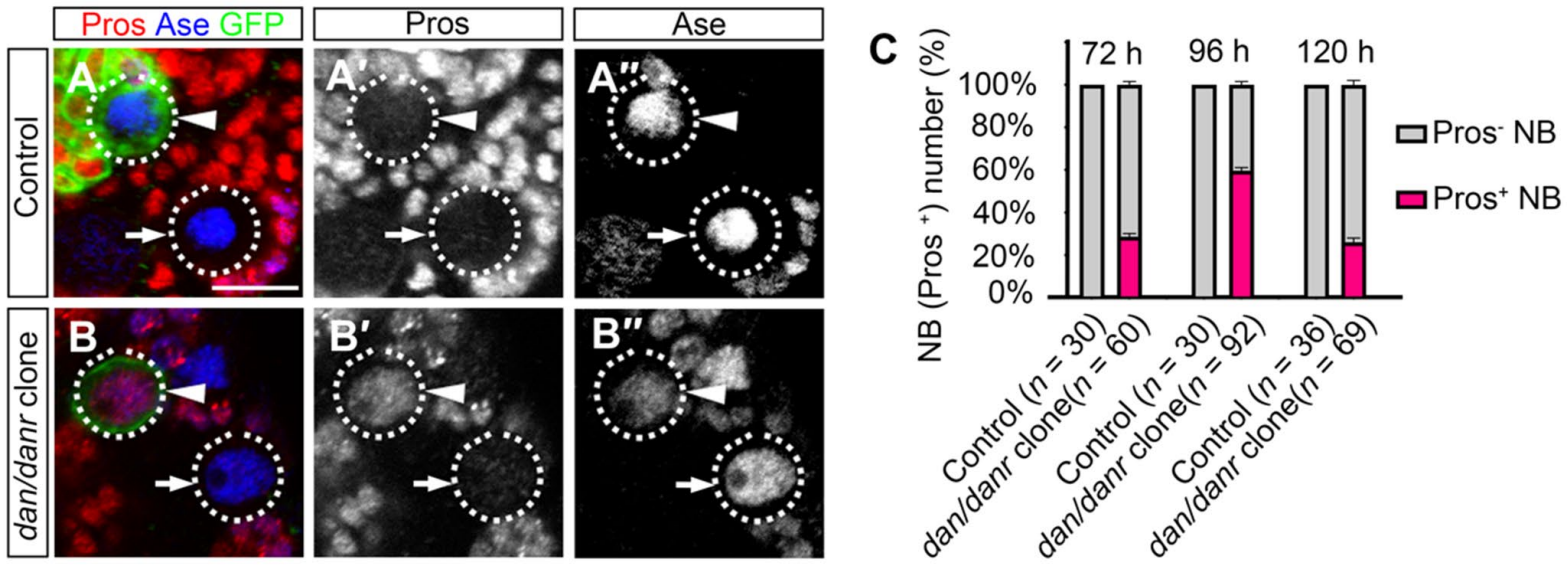
Loss of Dan/Danr leads to nuclear Pros in NBs. **(A–B″)** Anti-Pros and anti-Ase double labeling in third instar larval brains. **(A–A″)** NBs in *wt* clone (arrowhead) and its neighborhood (arrow) fail to show Pros in nuclei. **(B–B″)** NBs in *dan/danr* clones (arrowhead) exhibit nuclear Pros (red), but not in the *wt* NBs (arrow) in its neighborhood. This indicates that the loss of Dan/Danr is responsible for the nuclear Pros in NBs. GFP (green) labels the MARCM clones. Scale bar:10 μm. **(C)** The quantifications of larval brain NBs with nuclear Pros at 72 h, 96 h and 120 h after larvae hatching (ALH). Data are presented as mean ± SEM. Three replicates were conducted in each group, about 10 cells/replicate in control and 20 to 30 cells/replicate in *dan/danr* clones. The total measured cell number were on shown on figure panel. The percentage of NBs containing nuclear Pros is to about 60% at 96 h ALH.

The observation of smaller lineages of *dan/danr* NB clones prompted us to examine Pros location in these NBs. Nuclear accumulation of Pros has been considered as a typical signal for the termination of NB cell fate (Lai and Doe, 2014; Wu et al., 2019; Sang et al., 2022). We found that in the *dan/danr* NBs (Figures 2A–C). Approximately 61% (*n* = 92) of the NBs in *dan/danr* MARCM clones presented with nuclear Pros at 96 h ALH, while no corresponding presence of Pros was observed in any of their counterparts in *wt* controls (*n* = 30). These observations suggest that lack of *dan/danr* leads to nuclear Pros accumulation in larval brain NBs.

We evaluated the time window of the appearance of nuclear Pros in *dan/danr* NBs during the development. *dan/danr* clones showed nuclear Pros in the NBs between 72 h to 120 h ALH, with a peak at 96 h ALH (Figure 2C). The frequencies of NBs with nuclear Pros were 28% (*n* = 60) at 72 h ALH, 61% (*n* = 92) at 96 h ALH, and 26% (*n* = 69) at 120 h ALH (Figure 2C). No NBs with nuclear Pros were observed in *wt* controls among these time periods. Based on those data we determine that *dan/danr* may act to prevent nuclear Pros between 72 h to 120 h ALH.

### 3.3. Mutation of *dan/danr* alters the NB cell cycle

**Figure 3:**
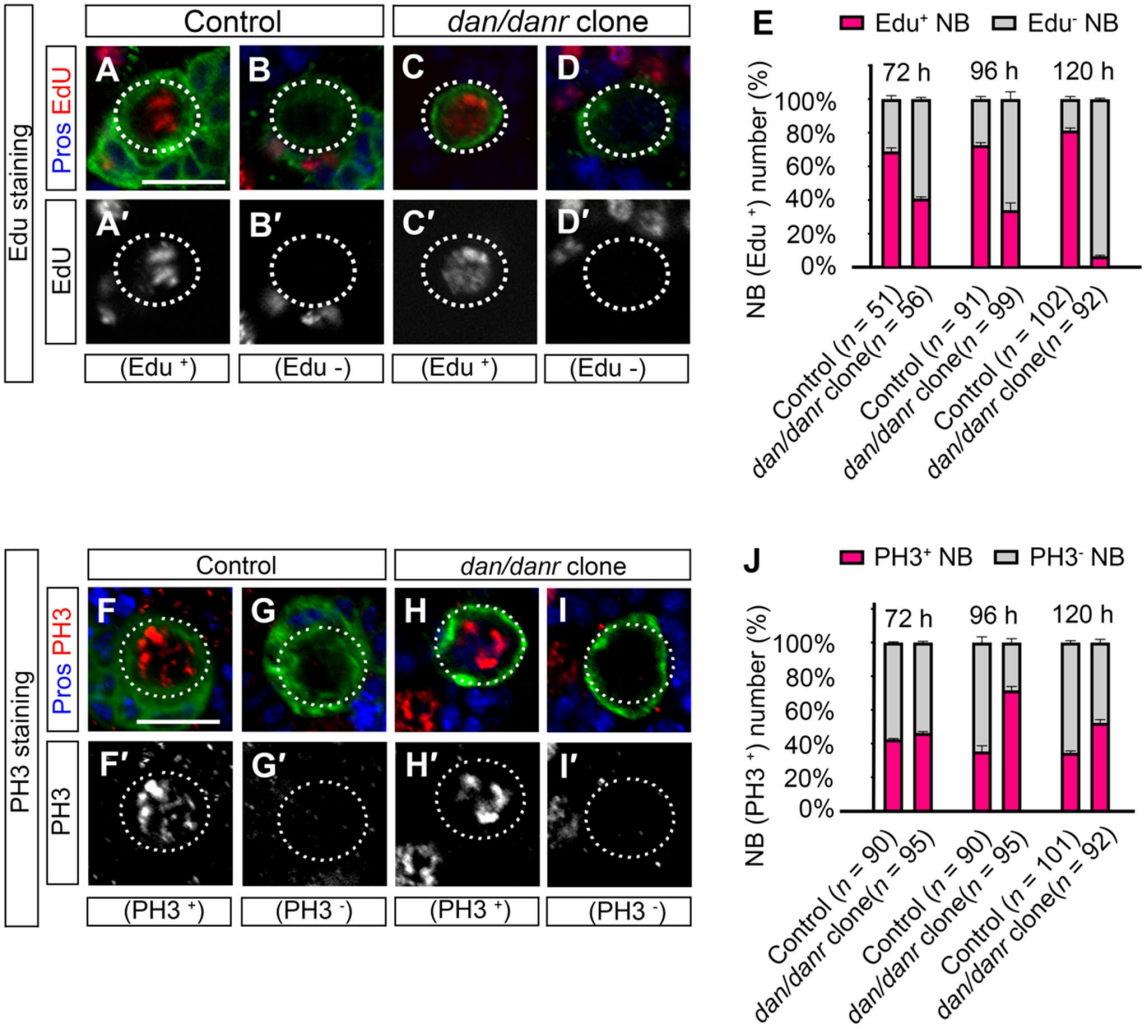
Mutations of *dan/danr* cause NB cell cycle defects in the third instar larval brains. **(A–D′)** Visualization of incorporated EdU in NBs of MARCM clones. EdU positive (red, **A,A’**, **C,C′**) and negative **(B,B′, D,D′)** NBs in the control and in *dan/danr* clones. **(E)** The quantitative data of Edu labeling in control and *dan/danr* NBs. Data are presented as mean ± SEM. Three replicates were conducted in each group, about 20 to 30 cells/replicate in each group. The total measured cell number were on shown on figure panel. The incorporation of EdU becomes less efficient with the time in *dan/danr* NBs. **(F–I′)** Anti-PH3 staining of NBs in MARCM clones. PH3 (red, **F,F′**, **H,H′**) positive and negative **(G,G’, I,I′)** NBs in the control and *dan/danr* clones. **(J)** The quantitative data of PH3 positive NBs in control and *dan/danr* clones. Data are presented as mean ± SEM. Three replicates were conducted in each group, about 30 cells/replicate in each group. The total measured cell number were on shown on figure panel. The percentages of PH3 positive NBs in *dan/danr* clones are higher than those of the control NBs, which show the largest differences at 96 h ALH. GFP (green) marks the MARCM clones. Dotted lines outline the NBs. Scale bars: 10 μm.

Previous studies have reported that NBs with nuclear Pros often exhibit cell cycle defects (Li and Vaessin, 2000; Wu et al., 2019; Liu et al., 2020). To determine the cell cycle progression of *dan/danr* NBs, we employed the Edu incorporation method and found that less NBs were Edu positive in *dan/danr* clones as compared with the controls at 96 h ALH (Figures 3A–E). Only 35% (*n* = 99) of NBs were labeled by Edu in *dan/danr* clones whereas the ratio of Edu positive NBs was almost 70% (*n* = 91) in the controls (Figure 3E). We noted that at 72 h ALH, the ratio of Edu positive NBs was also lower in *dan/danr* clones, being about 41% (*n* = 56) compared to 68% (*n* = 51) in the controls (Figure 3E). At 120 h ALH, it was only 7% (*n* = 92) in *dan/danr* clones but 82% (*n* = 102) in the controls (Figure 3E).

We also performed immunofluorescence staining with another cell cycle marker, PH3, at different developmental stages (Figures 3F–J). Unexpectedly, we found that more NBs (71%, *n* = 95) were PH3 positive in *dan/danr* clones as compared with the controls (35%, *n* = 90) at 96 h ALH (Figure 3J). We also checked the NBs at 72 h and 120 h ALH (Figure 3J). The results showed that the PH3 positive frequencies were also higher in *dan/danr* NBs being 46% (*n* = 95) at 72 h ALH, and 52% (*n* = 92) at 120 h ALH in *dan/danr* clones compared to 42% (*n* = 90) at 72 h ALH and 35% (*n* = 101) at 120 h ALH in the controls. Our Caspase-3 staining results also clarified that no apoptosis had occurred in the *dan/danr* clones (Supplementary Figure S2). These observations suggest that in the absence of *dan/danr*, the M-phase of the NB is prolonged, leading to an overall elongated cell cycle.

### 3.4. The expressions of Ase and Dpn are inhibited in *dan/danr* NBs

**Figure 4:**
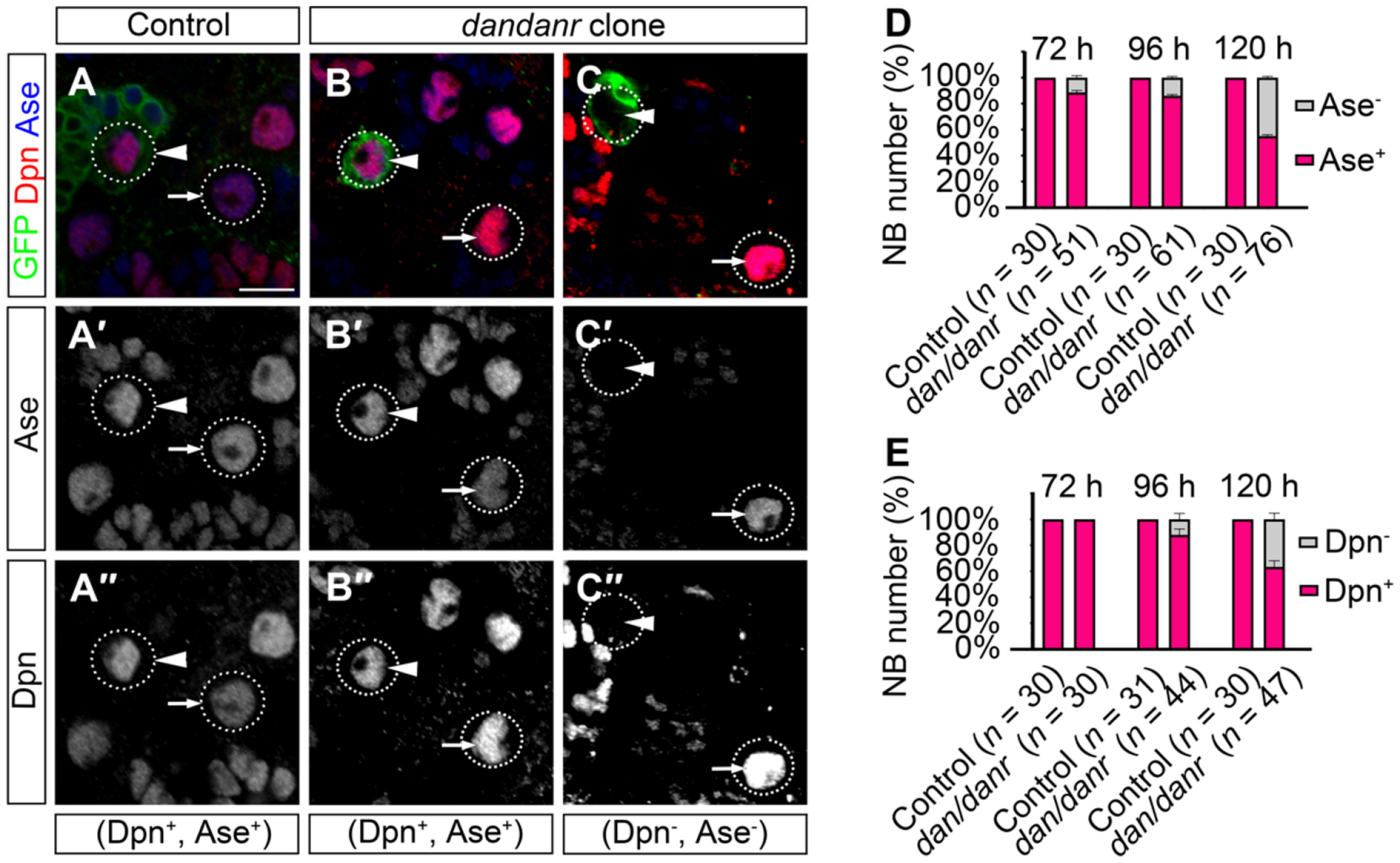
Expressions of Ase and Dpn are suppressed in *dan/danr* NBs. **(A–C″)** Anti-Ase (blue) and anti-Dpn (red) double labeling of NBs in MARCM clones in third instar larval brains. NBs in *wt* clone (arrowhead) and its neighbor (arrow) show as Ase and Dpn positive **(A–A″)**, while NBs in *dan/danr* clones present with two types: Ase and Dpn positive NBs (arrowhead, **B–B″**) or Ase and Dpn negative (arrowhead, **C–C″**). Note the *wt* NBs outside the clones (arrow, **B–C″**) are Ase and Dpn positive. This suggests that the expressions of Ase and Dpn are suppressed in *dan/danr* NBs. GFP (green) marks the clones. Scale bar:10 μm. **(D,E)** The statistical data of Ase **(D)** and Dpn **(E)** expressions in NBs of both control and *dan/danr* clones at 72 h, 96 h and 120 h ALH. Data are presented as mean ± SEM. Three replicates were conducted in each group, about 10 cells/replicate in each group. The total measured cell number were on shown on figure panel. In *dan/danr* MARCM clones, Ase and Dpn expressions are gradually inhibited in NBs. The ratio of NBs that are Ase and Dpn negative rises up to 40% in late larval brains (120 h ALH).

NB terminating features include the presence of nuclear Pros and the loss of NB markers (Maurange et al., 2008; Chai et al., 2013; Wu et al., 2019). We examined the cell fate identity of NBs using anti-Ase and anti-Dpn, as two markers for type I NBs. The absence of either marker expression would indicate a change of NB cell fate. As shown in Figure 4A, the large cells with Ase positive (~10 μm) were type I NBs, while 11% (*n* = 51) of the large cells in *dan/danr* MARCM clones were not labeled by anti-Ase at 72 h ALH (Figures 4A–D). This ratio further increased from 72 h to 120 h ALH (Figure 4D). About 15% (*n* = 61) of NBs were shown to be Ase negative at around 96 h ALH, with the percentage being 44% at 120 h AHL (*n* = 76) (Figure 4E). However, in *wt* controls all type I NBs were Ase positive (*n* = 30). This observation suggests that the NBs had gradually lost their NB identity in *dan/danr* mutant clones. Meanwhile, Dpn immunofluorescence staining showed a similar tendency in that the ratio of Dpn negative NBs increased from 72 h ALH to 120 h ALH in *dan/danr* clones (Figures 4A–C,E).

### 3.5. Nuclear Pros disrupts cell fate maintenance in *dan/danr* NBs

**Figure 5:**
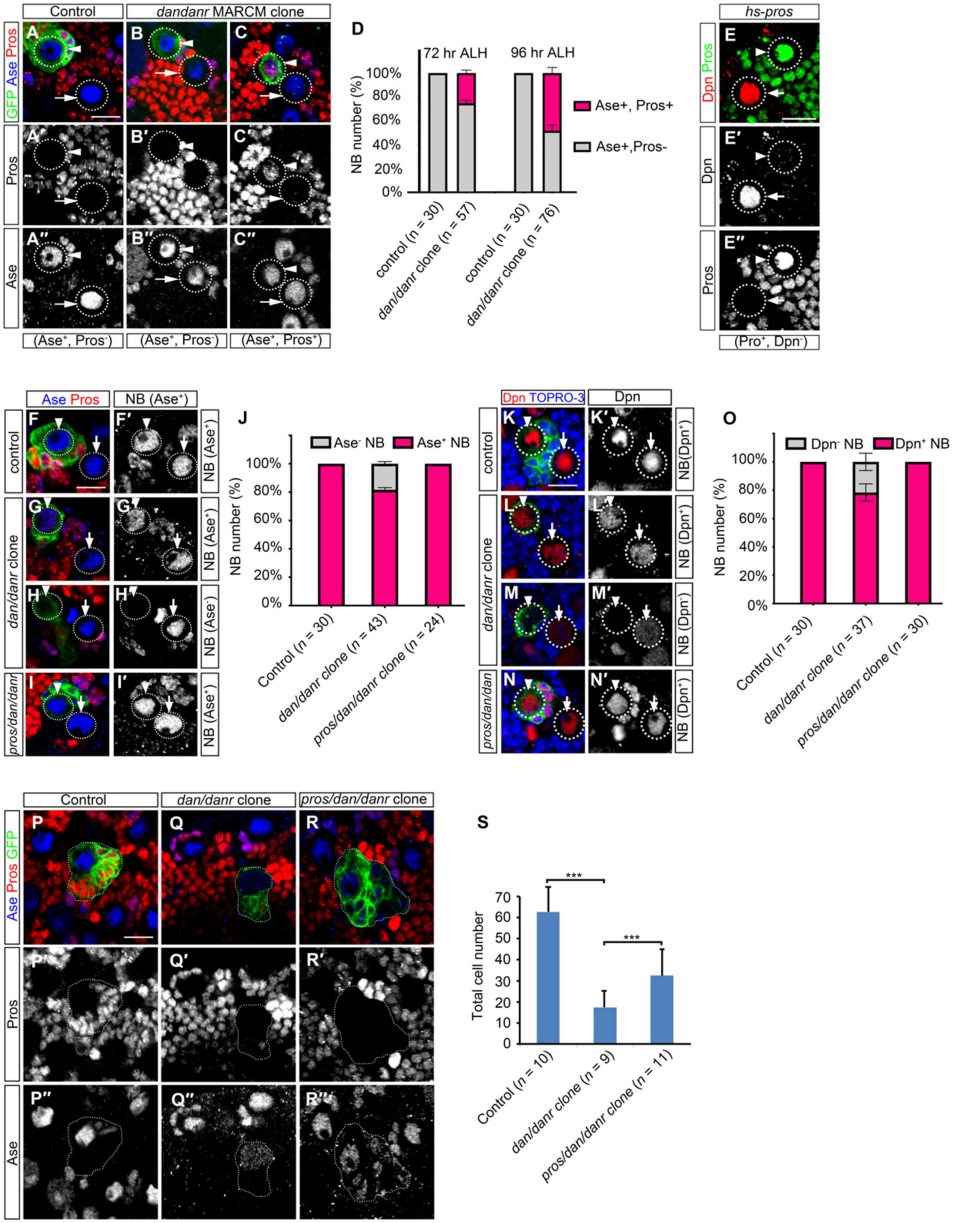
Nuclear Pros disrupts NB cell fates in *dan/danr* NBs in third instar larval brains. **(A–C″)** Anti-Pros (red) and anti-Ase (blue) double labeling of NBs in MARCM clones. **(A–A″)** Type I NBs in control clones (arrowhead) and in the neighborhood (arrow) are Ase positive (blue) and nuclear Pros negative. **(B–C″)** NBs in *dan/danr* clones show up in one of two groups: either Ase positive (blue) and nuclear Pros negative (arrowhead, **B–B″**), or Ase positive (blue) and nuclear Pros positive (red) (arrowhead, **C–C″**). Note the *wt* NBs outside the clones (arrows, **B–C″**) are Ase positive and nuclear Pros negative. **(D)** Statistical data shows both Ase and nuclear Pros positive NBs in control and *dan/danr* clones at 72 and 96 h ALH. Data are presented as mean ± SEM. Three replicates were conducted in each group, about 10 cells/replicate in control and about 20 cells/replicate in *dan/danr* clones. The total measured cell number were on shown on figure panel. It seems that the appearance of nuclear Pros occurs earlier than the disappearance of Ase expression. **(E–E″)** Anti-Pros (green) and anti-Dpn (red) double staining of the heat-shock treated NBs. Heat shock induced Pros causes nuclear Pros accumulation (green, **E,E″**) and inhibits Dpn expression in NBs (arrowhead). The NBs (arrows) without nuclear Pros are Dpn positive (red, **E,E′**). This suggests nuclear Pros inhibits Dpn expression in NBs. **(F–I′):** Anti-Pros (red) and anti-Ase (blue) double labeling of the type I NBs in MARCM clones. **(F,F′)** Type I NBs in *control* clones (arrowhead) and outside clones (arrow) show as Ase positive (blue). **(G–H′)** Two types of NBs in *dan/danr* clones are identified: Ase positive (blue, arrowhead, **G,G′**) or Ase negative (arrowhead, **H,H′**). **(I,I′)** NBs in *pros/dan/danr* triple mutant clones (arrowhead) are Ase positive. Note the NBs outside the clones (arrow, **G–I′**) are *wt.* **(J)** The statistical data of As positive NBs in different genotypic backgrounds (*control, dan/danr, pros/dan/danr*). Data are presented as mean ± SEM. Three replicates were conducted in each group, about 10 cells/replicate in each group. The total measured cell number were on shown on figure panel. In *dan/danr* clones about 20% NBs fail to show Ase expression. This phenotype is reversed in *pros/dan/danr* triple mutant clones, indicating that suppression of Ase is due to nuclear Pros in *dan/danr* NBs. **(K–N′)**: Anti-Dpn (red) staining of the NBs. **(K,K′)** NBs in *control* clones (arrowhead) and its neighbors (arrow) are Dpn positive. **(L–M’)** Two types of NBs in *dan/danr* clones are observed: Dpn positive (red, arrowhead, **L,L′**) and Dpn negative (arrowhead, **M,M′**). **(N,N′)** NB in *pros/dan/danr* triple mutant clones (arrowhead) is Dpn positive. Please note that the NBs outside the clones (arrow, **K–N′**) are *wt.* **(O)** Statistical data of Dpn negative NBs in different genotypes (*control, dan/danr, pros/dan/danr*). Data are presented as mean ± SEM. Three replicates were conducted in each group, about 10 cells/replicate in each group. The total measured cell number were on shown on figure panel. In *dan/danr* clones about 20% NBs fail to show Dpn expression and this phenotype is reversed in *pros/dan/danr* triple mutant clones. This indicates that, in a similar observation to Ase, suppression of Dpn is due to nuclear Pros in *dan/danr* NBs. **(P–R)** The sizes of MARCM clones of different genotypes (**P**: *control*, **Q**: *dan/danr*, **R**: *pros/dan/danr*). **(S)** The statistical data of average total cell numbers in clones of different genotypes. Unpaired student t-test was performed (***p < 0.001). The measured clone number was shown on figure panel. The *dan/danr* clone sizes are partially rescued by the removal of Pros (*pros/dan/danr*). GFP (green) marks MARCM clones. Circular dotted lines outline NBs. Dotted lines outline MARCM clones. TO-PRO-3 (Blue) labels DNA. Scale bars: 10 μm.

We double labeled NBs in *dan/danr* clones with Ase and Pros at different time points. The ratios of nuclear Pros and Ase double positive NBs increased from 72 h to 96 h ALH (Figures 5A–D), from 25% (*n* = 57) to 58% (*n* = 76), respectively. This suggests that the appearance of nuclear Pros occurs earlier than the disappearance of Ase expression. It is known that heat-shock induced overexpression of Pros in *wt* NBs leads to nuclear Pros accumulation (Choksi et al., 2006; Lai and Doe, 2014). We also observed that nuclear Pros from heat-shock induced expression inhibited the stem cell marker Dpn expression in larval brain NBs (Figure 5E).

We then removed Pros from *dan/danr* NBs by producing a *pros*/*dan/danr* triple mutant MARCM clone. We found that the Ase or Dpn expression phenotype in mutant NBs were rescued (Figures 5F–O). Ase immunofluorescence staining data showed that all NBs (*n* = 24) showed Ase positive in *pros/dan/danr* triple mutant clones, whereas there were about 82% (*n* = 43) NBs showing Ase positive in their *dan/danr* double mutant counterparts (Figures 5F–I,J). Dpn staining also revealed similar results. All NBs (*n* = 30) exhibited Dpn expression in *pros/dan/danr* clones, while only 82% (*n* = 37) NBs showed Dpn in the *dan/danr* NB clones (Figures 5K–N,O). Furthermore, the clone sizes of *pros/dan/danr* NB were larger than those of *dan/danr* clones (Figures 5P–S). There were 32 ± 8 (*n* = 11) cells in each *pros/dan/danr* clone, whereas only 17 ± 8 (*n* = 9) cells in each of the *dan/danr* clones (*p* = 0.009) (Figure 5S). These observations suggest that nuclear Pros suppresses Ase and Dpn expression in *dan/danr* NBs and the removal of nuclear Pros rescues not only Ase and Dpn expression but also the NB lineage number.

### 3.6. Dan/Danr and nuclear Pros are mutually exclusive in NBs

**Figure 6:**
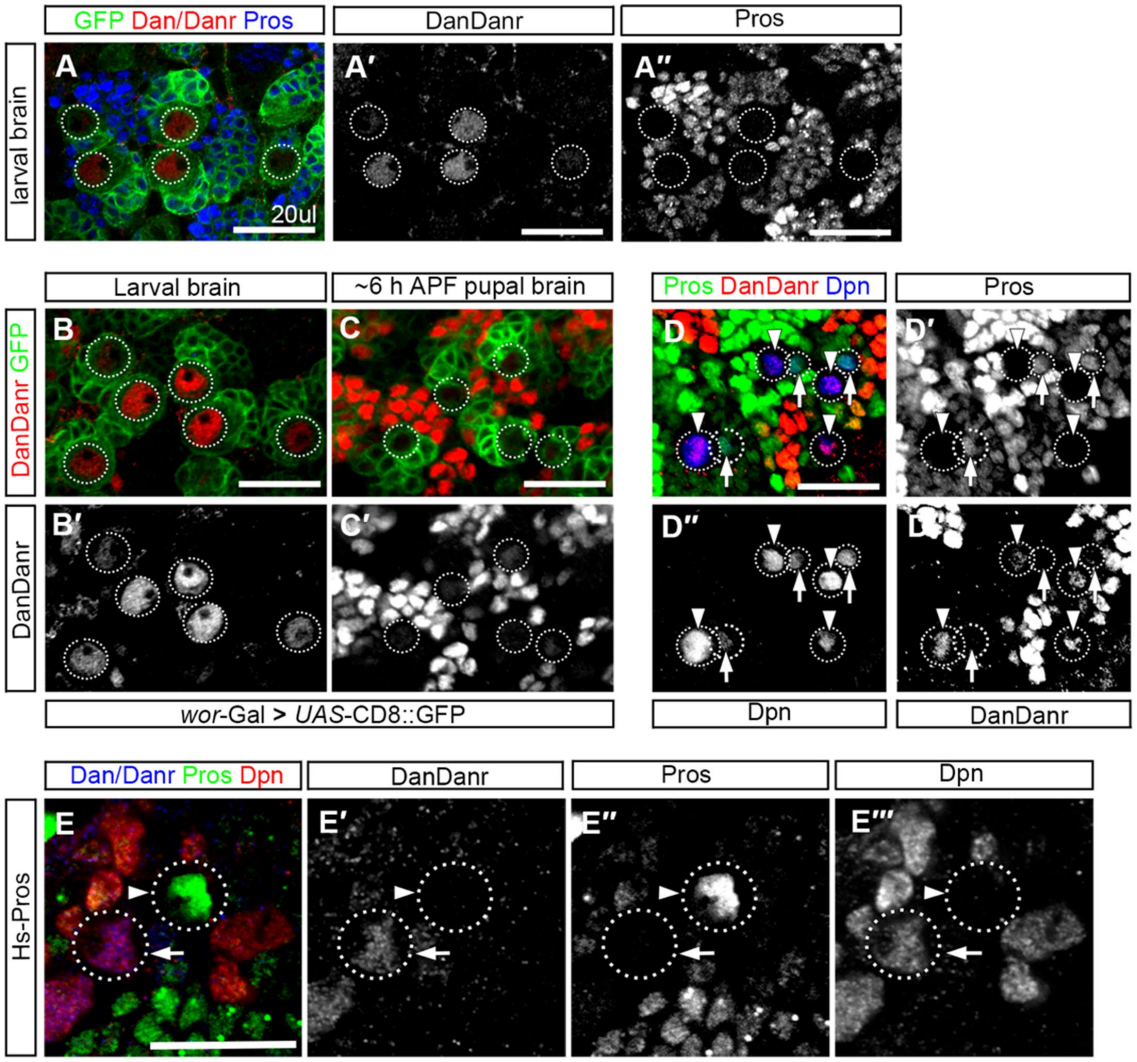
Dan/Danr and nuclear Pros are mutually exclusive in NBs. **(A–A″)** Anti-Dan/Danr and anti-Pros double labeling of the NBs in third instar larval brains. Dan/Danr (red, **A,A′**) are detected in NBs, while nuclear Pros (blue, **A,A″**) remains undetectable. Both of them do not coexist in NBs. GFP (green) derived by ase-Gal4 marks NBs and their progeny. Dotted lines outline the NBs. Scale bar: 20 μm. **(B–C′)** Anti-Dan/Danr (red) staining of the NBs between larval to early pupal stage (6 h after pupae formation, APF). *dan/danr* is easily detected in larval brain NBs (red, **B,B′**), but is barely detected in pupal brain NBs **(C,C′)**. This suggests that Dan/Danr expression level is decreased in pupal NBs. Note that NBs become smaller in early pupal brains. GFP (green) derived by *ase* - Gal4 marks NBs and their progeny. Dotted lines outline the NBs. Scale bar: 20 μm. **(D–D″′)** Anti-Dpn (blue), anti-Dan/Danr (red) and anti-Pros (green) triple staining of NB in early pupal stage (~16 h APF). Two type NBs are detected: Dpn+, Dan/Danr +, nuclear Pros – NBs (Arrowheads) and Dpn+, Dan/Danr -, nuclear Pros + NBs (Arrows). Dpn positive cells are NBs. Dotted lines outline the NBs. The scale: 20 μm. **(E–E″)** Anti-Pros (green), anti-Dan/Danr (blue), and anti-Dpn (red), triple staining of the heat shock treated NBs. Heated shock induced Pros leads to nuclear Pros (arrowhead, green) in NBs and prevents Dan/Danr expression. The NBs without nuclear Pros show Dan/Danr (arrow, blue). This observation indicates that nuclear Pros and Dan/Danr are mutually exclusive in NBs. Dotted lines outline the NBs. Scale bar: 40 μm.

To understand the expression pattern of *dan/danr* at the early pupal stage when nuclear Pros appears in NBs prior to their termination. We firstly performed Dan/Danr and Pros double labeling in the *wt* third instar larval brains and found that Dan/Danr were present in the NB nuclei and that no nuclear Pros was detectable (Figure 6A). We then carried out Dan/Danr staining at late larval and early pupal stages. We found that at the early pupal stage (6 h APF) the fluorescent signals of anti-Dan/Danr in NBs were weaker as compared with the ones at third instar larval stage (Figures 6B,C). And nuclear Pros was largely undetected even the cell sizes of NBs were smaller. In order to examine the expression pattern between Dan/Danr and Pros in terminating NBs, Dpn was used as NB marker and older pupal brains (16 h APF) were adopted. Dpn, Dan/Danr and Pros triple-labeled experimental data showed that two groups of Dpn positive NBs were identified (i) Dan/Danr positive, nuclear Pros negative, and (ii) Dan/Danr negative, nuclear Pros positive (Figure 6D). Since these *wt* NBs were in a sequential process of NB termination, Dan/Danr negative/nuclear Pros positive NBs should represent the last state before terminal division. Thus, as Dan/Danr positive, nuclear Pros negative NBs are prior to Dan/Danr negative/nuclear Pros positive NBs, it is logical to deduct that Dan/Danr prevents nuclear Pros in NBs. At the time when Dan/Danr expression are lost, nuclear Pros appears in NBs, which leads to the termination of NBs.

We also performed Pros over-expression experiments in the *wt* third instar larval brains. Double-labeled experiments indicate that heat-shock induced Pros in NBs led to nuclear Pros and that Dan/Danr were not detectable in these NBs with nuclear Pros (Figure 6E). This suggests that the nuclear Pros alone is sufficient to inhibit Dan/Danr expression. At early pupal stage Dan/Danr expression are decreased and nuclear Pros appears in NBs. We further induced Flip-out clones of UAS-Dan. Results showed that Pros is decreased, where Dan/Danr immunostaining were increased in the clones, (Supplementary Figure S3). Thus, Dan/Danr and Pros are mutually exclusive in NBs.

### 3.7. Dan/Danr are partially required for Grh expression to control NBs size

**Figure 7:**
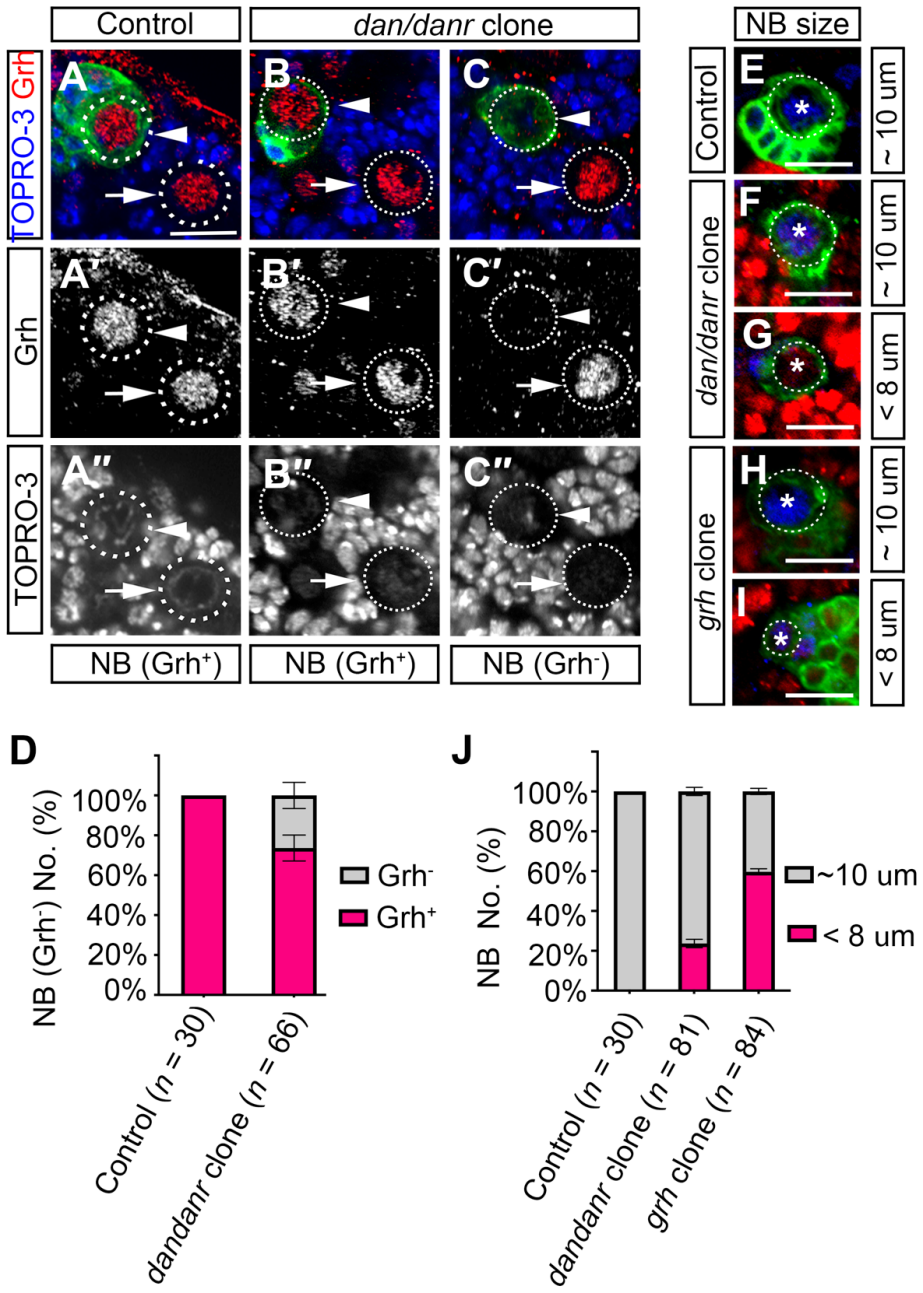
Dan/Danr are partially required for Grh expression in NBs cell size controlling. **(A–C″)** Anti-Grh (red) staining of NBs in *dan/danr* MARCM clones of third instar larval brains. **(A–A″)** NBs in control clones (arrowhead) and in the neighborhood (arrow) show as Grh (red) positive. **(B–C″)** In *dan/danr* clones, two types of NBs are detected: Grh (red) positive NBs (**B–B″**, arrowhead) and Grh negative NBs (**C–C″**, arrowhead). Their neighboring NBs (arrow) are *wt* and show as Grh (red) positive. GFP (green) labels the clones and TO-PRO-3 (blue) marks the DNA. Scale bar: 20 μm. **(D)** The statistical data of Grh negative NBs in *dan/danr* clones at 96 h ALH. Data are presented as mean ± SEM. Three replicates were conducted in each group, about 10 cells/replicate in control and about 22 cells/replicate in *dan/danr* clones. The total measured cell number were on shown on figure panel. About 20% of *dan/danr* NBs are Grh negative. This indicates that *dan/danr* are partially required for Grh expression in NBs. **(E–I)** The NBs cell sizes in *dan/danr* and *grh* MARCM clones at late larval stage. GFP (green) labels the control **(E)**, *dan/danr* **(F,G)** and *grh* **(H,I)** clones. Ase (blue) marks the NBs. In control clones, the NB is around 10 μm. But in both of *dan/danr* and *grh*, smaller (< 8 μm) NBs are observed **(G,I)**. Scale bars: 10 μm. **(J)** The statistical data of NBs cell sizes in control, *dan/danr* and *grh* clones at 96 h ALH. Data are presented as mean ± SEM. Three replicates were conducted in each group, about 10 cells/replicate in control and about 20 cells/replicate in *dan/danr* or *grh* clones. The total measured cell number were on shown on figure panel. It is possible that the loss of Grh expression in *dan/danr* NBs leads to smaller NBs cell sizes.

The observations that lack of Dan/Danr leads to the appearance of nuclear Pros and changes of NB cell fate between 72 h to 120 h ALH, seemed reminiscent of aspects of NB temporal regulation. As Cas and Svp are early temporal regulators which are expressed before the third instar larval brain NBs (Jacob et al., 2008). We examined whether *cas* or *svp* affects *dan/danr* expression. Immunofluorescence data showed that Dan/Danr protein levels did not change in *cas* or *svp* MARCM clone NBs (Supplementary Figure S4). This suggests that *dan/danr* expression did not depend on the early temporal regulators Cas and Svp. The temporal regulator Grh functions in the late stage of NBs temporal regulation (Cenci and Gould, 2005; Chai et al., 2013), we examined *grh* expression in *dan/danr* NBs by fluorescence staining (Figures 7A–C). About 26% (*n* = 66) of the *dan/danr* NBs failed to express Grh at 96 h ALH (Figure 7D). These data suggest that Dan/Danr are also required for Grh expression in the third instar larval brain NBs.

To further understand the relationship between Dan/Danr and Grh, we approached *grh* MARCM clones in larval brains and found that the cell sizes of NB became smaller and that Pros accumulated in NB nuclei (Figures 7E–J; Supplementary Figures S5A–D). About 60% (*n* = 84) of the NBs exhibited smaller cell sizes (< 8 μm) and about 29% (*n* = 79) of the NBs showed nuclear Pros in *grh* MARCM clones at 96 h ALH (Figure 7J; Supplementary Figure 5D). The quantification of NB cell sizes in *dan/danr* NBs showed that about 24% (*n* = 81) of NBs displayed smaller cell sizes (< 8 μm) in *dan/danr* clones at 96 h ALH (Figures 7E–J). Since this ratio was similar to the percentage of NBs that had failed to express *grh* in *dan/danr* clones, it is possible that the loss of Grh in *dan/danr* NBs is responsible for the NB cell size change.

## 4. Discussion

Sustained neural stem cell proliferation plays a vital role in neurogenesis. Our work shows that the *Drosophila* pipsqueak domain transcription factors Dan and Danr are required for NB cell fate maintenance. The transcription factors Dan/Danr have been reported to be involved in the embryonic development of both the eye and the CNS (Emerald et al., 2003; Curtiss et al., 2007; Kohwi et al., 2011). Here we found that *dan/danr* are expressed in the larval brain NBs. In the absence of Dan/Danr, NBs produce less progenies and their lineages become smaller.

The absence of Dan/Danr could lead to three events in the larval brain NBs: (i) Pros accumulation in nuclei; (ii) loss of NB markers (Ase and Dpn); and (iii) cell cycle alteration. Among these, NB molecular marker loss and cell cycle alteration are the consequence of the presence of nuclear Pros in *dan/danr* NBs. It has been reported that heat-shock induces the overexpression of Pros and that this results in nuclear Pros accumulation and the suppression of the expression of the NB markers Ase and Dpn (Choksi et al., 2006; Lai and Doe, 2014). Our data support the hypothesis that, in the absence of Dan/Danr, Pros enters the nuclei and then suppresses the expression of NB markers Ase and Dpn. We have shown that Pros appears in the nuclei in the majority of NBs prior to Ase fade-away at 96 h ALH. In addition, all NBs retain Dpn and Ase expressions when *pros* is removed from *dan/danr* clones, and the removal of *pros* in *dan/danr* NBs partially rescues the NB lineage size shrinkage phenotype. This indicates that nuclear Pros is also partially responsible for the cell cycle alteration in *dan/danr* NBs. This is consistent with the previous reports that Pros regulates not only NB marker expressions but also cell cycle progression (Li and Vaessin, 2000; Lai and Doe, 2014). Thus, we propose that Dan/Danr act to prevent premature nuclear Pros, cell cycle defects, and NB cell fate changes.

At the larval stage, Dan/Danr are mainly expressed in NBs while nuclear Pros remains undetected. At the early pupal stage, Dan/Danr are decreased in NBs, just at the time point that Pros signals begin to present themselves in the nuclei of the NBs. In our confocal images, Dan/Danr positive NBs failed to show nuclear Pros signals, and vice versa. Premature nuclear Pros is only detected in *dan/danr* mutant NBs and the overexpression of Pros in *wt* NBs results in the detection of nuclear Pros and suppression of Dan/Danr. These observations suggest that Dan/Danr and nuclear Pros appear to antagonize each other and are mutually exclusive in NBs.

Our previous study reported that the lack of RanGap facilitates nuclear Pros accumulation in NBs (Wu et al., 2019). In order to further uncover the relationship between *dan/danr* and nuclear Pros, we checked RanGAP expression in *dan/danr* NBs. We discovered that Dan/Danr is expressed in *rangap* NBs in the larval brains. While in *dan/danr* NBs, the RanGAP was absent (Supplementary Figure S6). We propose that Dan/Danr and nuclear Pros appear to antagonize each other *via* RanGAP. However, neither RanGAP, nor Pros is regulated by Dan/Danr, and other pathway(s) might be involved in this process.

Grh is a late temporal regulatory factor and acts to prevent premature termination of NBs (Cenci and Gould, 2005; Chai et al., 2013). We show that in *grh* MARCM clones, about 60% of the NBs exhibited smaller cell sizes. The Grh expression partially depends on Dan/Danr. In *dan/danr* clones, the ratio of Grh negative NBs is about 26%. This coincides with the percentage of NBs displaying smaller cell sizes. This may reflect that Grh operates genetically downstream of Dan/Danr and acts to prevent NB cell size shrinkage.

**Figure 8:**
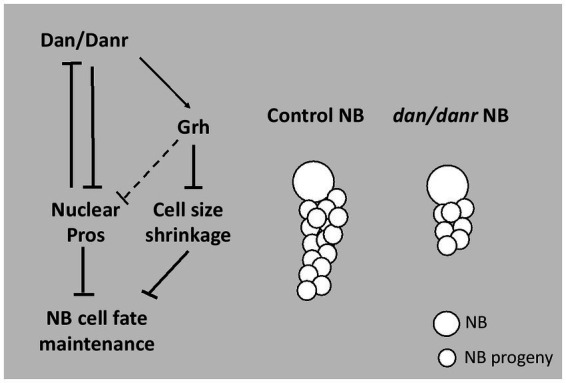
Diagram depicting Dan/Danr function. Dan/Danr antagonizes nuclear Pros to maintain NB cell fate in *Drosophila* third instar larval brains. During larval brain development, NBs maintain precise cell fate and size to form the central nervous system. In this process, *Drosophila* pipsqueak domain transcription factors, Dan/Danr act to antagonizes nuclear Pros in NBs and maintain NB cell fate. Dan/Danr are partially required for Grh expression to prevent the shrinkage of NB cell sizes. In addition, lack of Dan/Danr causes defective NB cell cycle progression, resulting in less progeny cells to result from NBs.

In conclusion, our study demonstrates a novel function of pipsqueak domain transcription factors Dan/Danr in NBs fate maintenance in the third instar larval brains (Figure 8). In the absence of Dan/Danr, premature nuclear Pros appears, leading to NB markers Ase and Dpn loss and smaller NB cell sizes.

## Data availability statement

The original contributions presented in the study are included in the article/Supplementary material, further inquiries can be directed to the corresponding authors.

## Author contributions

HA, YX, and YC conceived the idea of the project and designed the experiments. HA performed the experiments contributed to data analysis. YY, XR, and MZ conducted the genetic experiments. FZ, HZ, and RS generated the antibodies. YB and TL contributed to project discussion and coordination. HA wrote the initial manuscript. YX revised the manuscript. All authors read and approved the final manuscript.

## Funding

This work was funded by the Chinese National Natural Science Foundation (32000506), The Youth Innovation Team of Shaanxi Universities, and the Natural Science Special Project of Shaanxi Education Department (18JK0919).

## Conflict of interest

The authors declare that the research was conducted in the absence of any commercial or financial relationships that could be construed as a potential conflict of interest.

## Publisher’s note

All claims expressed in this article are solely those of the authors and do not necessarily represent those of their affiliated organizations, or those of the publisher, the editors and the reviewers. Any product that may be evaluated in this article, or claim that may be made by its manufacturer, is not guaranteed or endorsed by the publisher.

## Footnotes

^1^ www.flybase.org
