# Supplementary material for: Pipsqueak family genes dan/danr antagonize nuclear Pros to prevent neural stem cell aging in Drosophila larval brains
Source: Front Mol Neurosci. 2023 May 17;16:1160222. doi: 10.3389/fnmol.2023.1160222 (PMC10231327; doi:10.3389/fnmol.2023.1160222)
Supplement: Supplementary file 1 [file Data_Sheet_1.docx]

Supplementary Material

Pipsqueak family genes *dan/danr* antagonize nuclear Pros to prevent neural stem cell aging in *Drosophila* larval brains

# Supplementary Figures


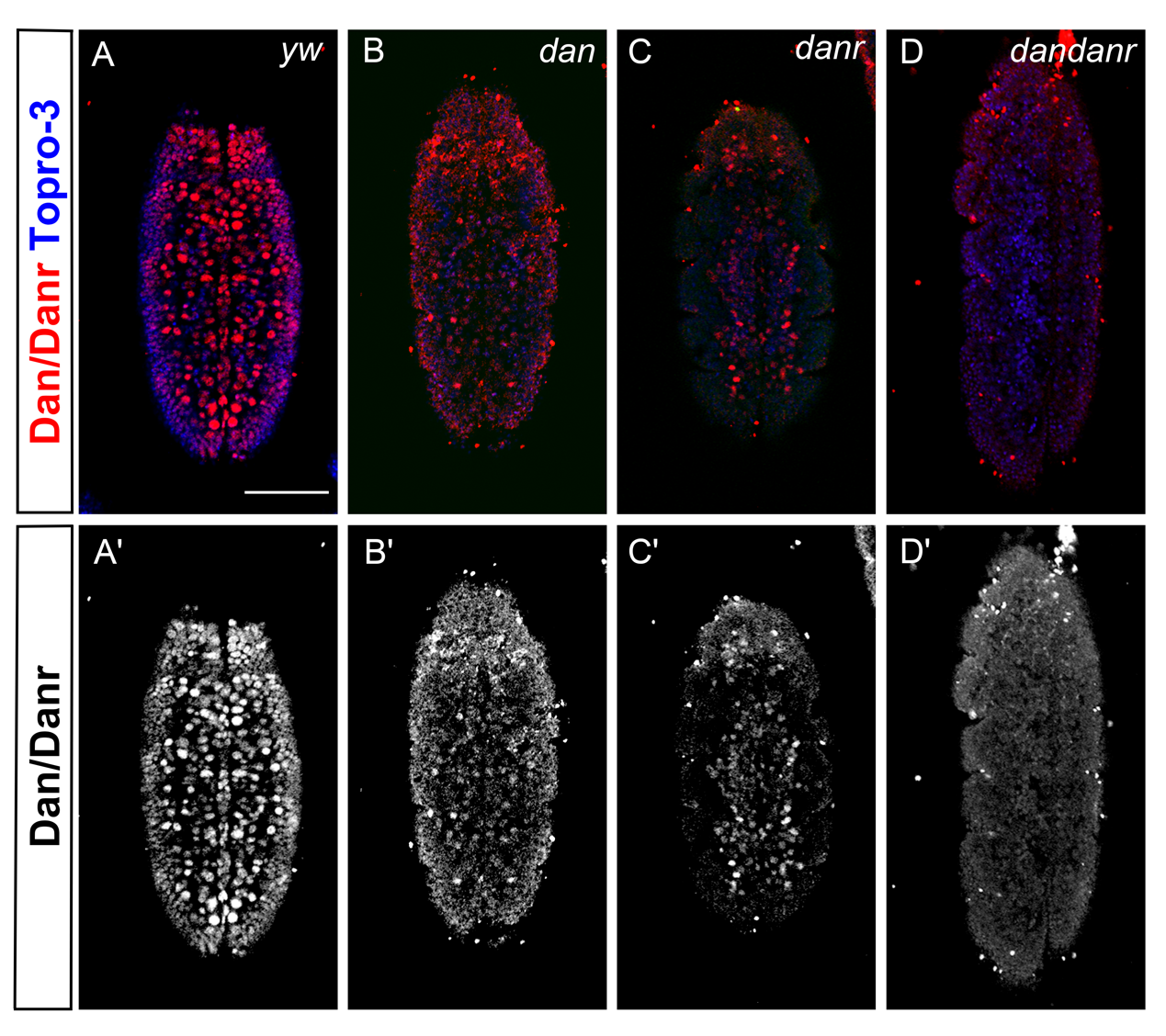


**Figure S1: Anti-Dan/Danr recognizes Dan and Danr in *Drosophila* NBs.**

**(A - D)** Anti-Dan/Danr (red) staining in different genotypes (*yw*, *dan*, *danr* and *dan/danr*) in the embryonic central nervous system. Anti-Dan/Danr signals are easily detected in embryonic NBs (A); Slight signals of anti-Dan/Danr are detected in both *dan* (B) and *danr* (C) single mutants due to *dan* and *danr* sharing a large proportion of their protein sequences. The signal of Dan/Danr is lost in the *dan/danr* (D) double mutant. This suggests the antibody of Dan/Danr is effective for Dan and Danr. TO-PRO-3 (blue) labels the DNA. Scale bar: 60 μm.


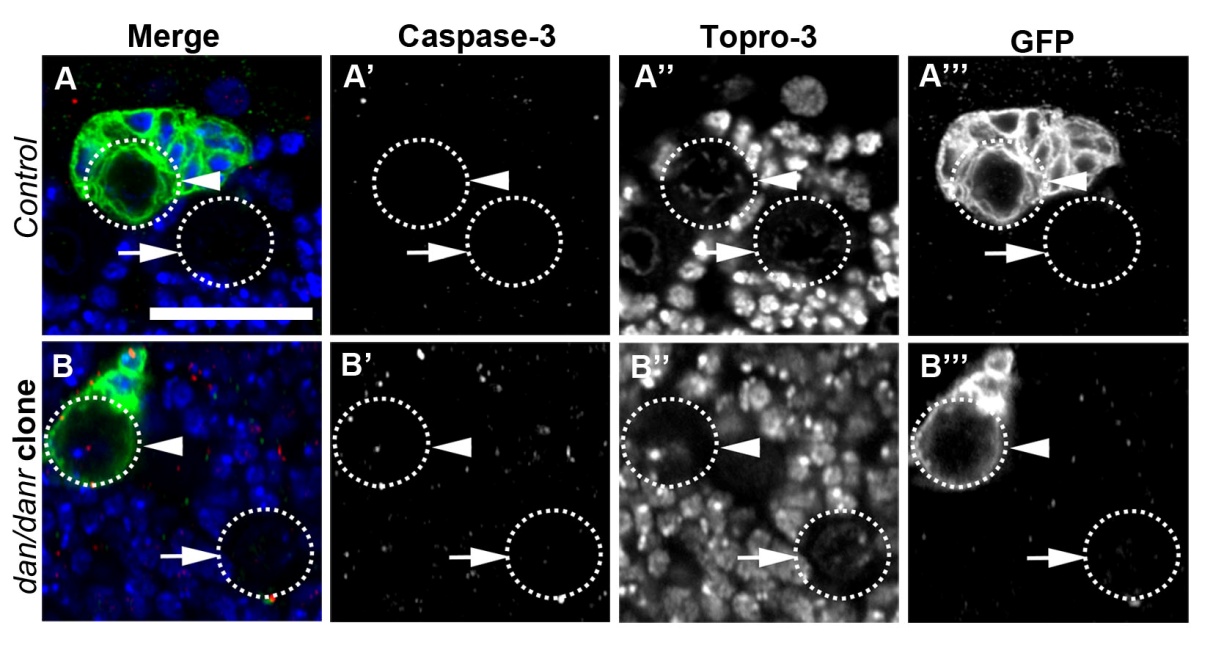


**Figure S2: Caspase-3 staining in *dan/danr* clones.**

The confocal pictures show the Caspase-3 (red) immunofluorescence staining in FRT control (A-A’’’) and *dan/danr* clone (B-B’’’). The clone was labeled by CD8::GFP (green) (A’’’ and B’’’) and DNA was labeled by TOPRO-3 (blue). Arrowheads indicate the NBs in the clones and arrows indicate the NB outside of the clones. Scale bar: 20 μm.


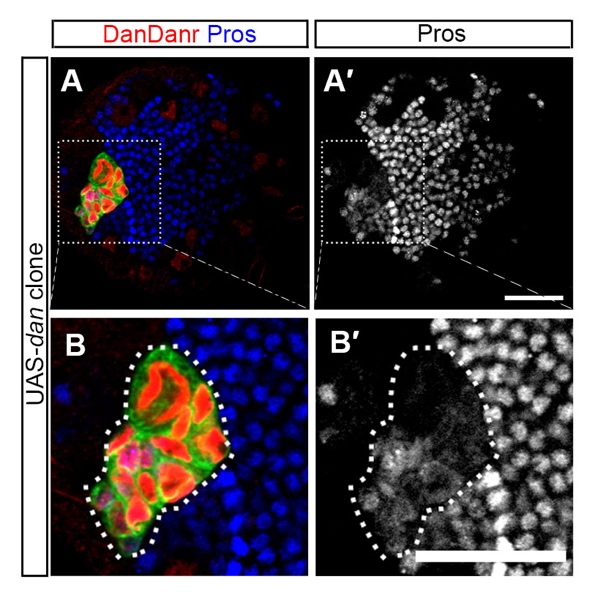


**Figure S3: Flp-out induced UAS-*dan* NB clones.**

Overexpression Dan/Danr (red) and lower Pros signals (blue) were observed in the clones A-A’ and zoomed image B-B’. GFP (green) and dotted lines label the NB clone. Scale bar: 40 μm.


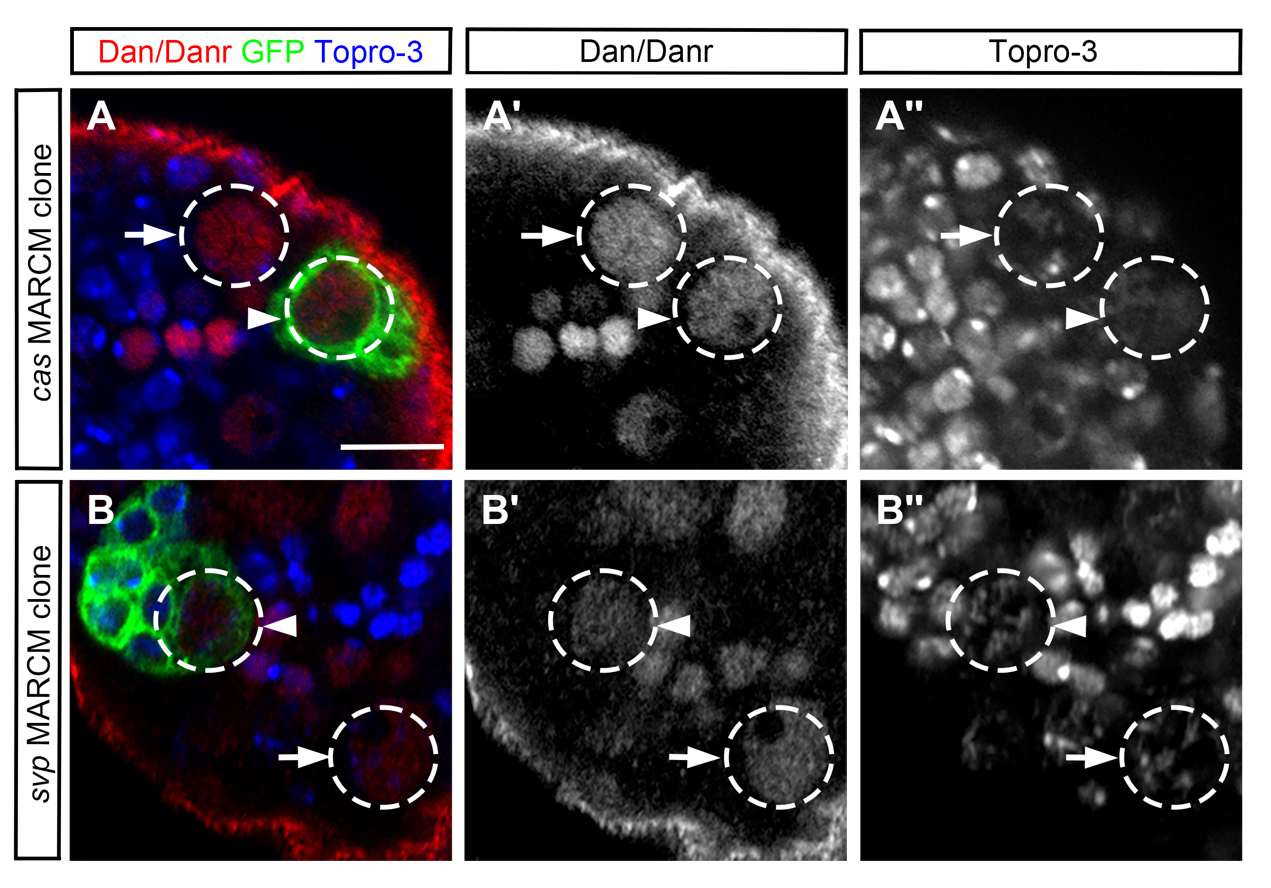


**Figure S4. Dan/Danr is not regulated by Cas and Svp in larval brain NBs.**

The NBs in *cas* (arrowhead, A-A'') and *svp* (arrowhead, B-B'') MARCM clones show the same level of anti-Dan/Danr signals (red) as their neighborhood controls (arrows, A-B'') at 72 hr ALH. This indicates that Dan/Danr expression does not depend on *cas* or *svp* gene expression. TO-PRO3 (blue) labels DNA to show the cell cycle phases. GFP (green) marks clones. Scale bar: 10 μm.


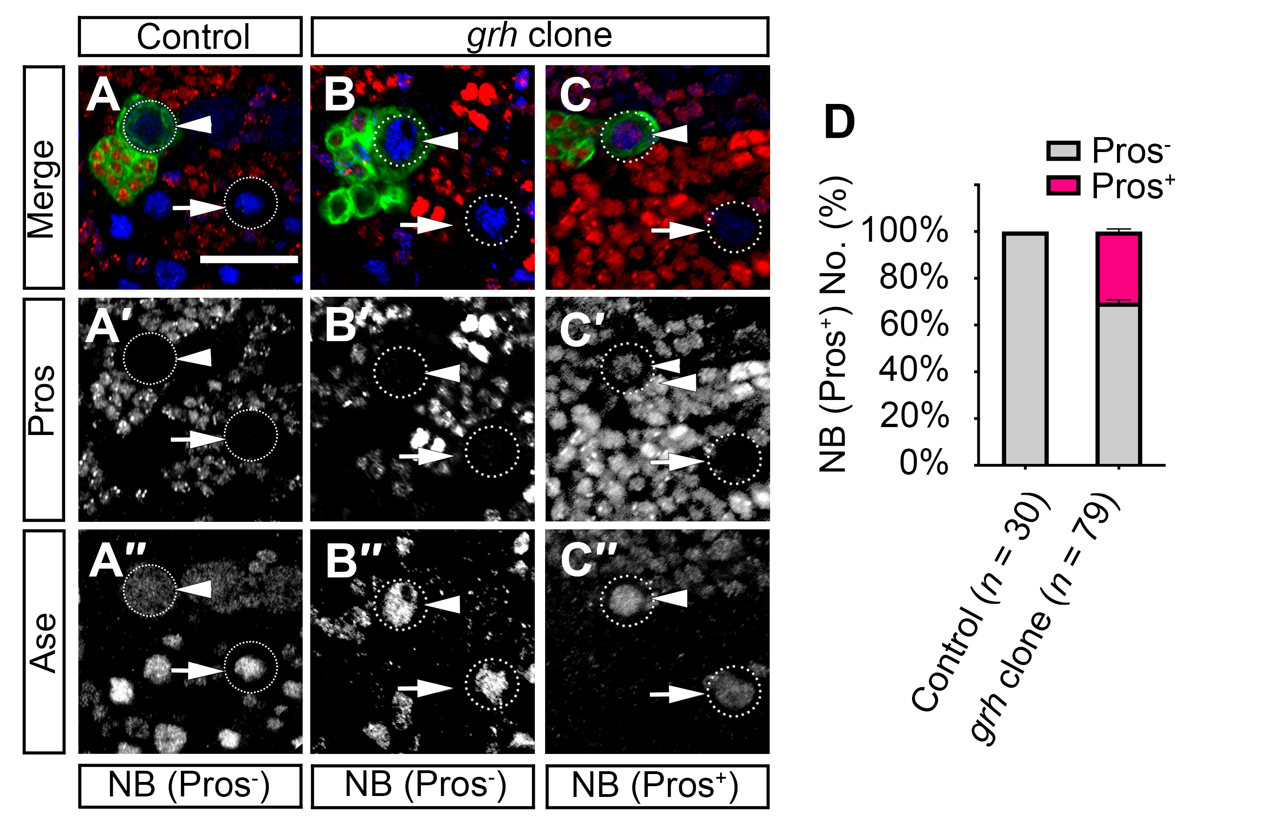


**Figure S5. Mutant of *grh* causes nuclear Pros in NBs at 120 hr ALH**

**(A-C'')** Anti-Pros (red) and anti-Ase (blue) double labeling of NBs in MARCM clones in late third instar brains. (A-A'') NBs in control clones (arrowhead) and in the neighborhood (arrow) show as Ase positive and nuclear Pros negative. (B-C'') NBs in *grh* clones show two types of NBs: Ase positive, nuclear Pros negative, and Ase and nuclear Pros double-positive. Note the NBs outside the *grh* clones are *wt*. GFP (green) labels the clones. Scale bar: 20 μm. **(D)** The ratio of NBs with nuclear Pros in *grh* clones is about 20%. This indicates that Grh is partially responsible for the prevention of nuclear Pros accumulation in NBs.


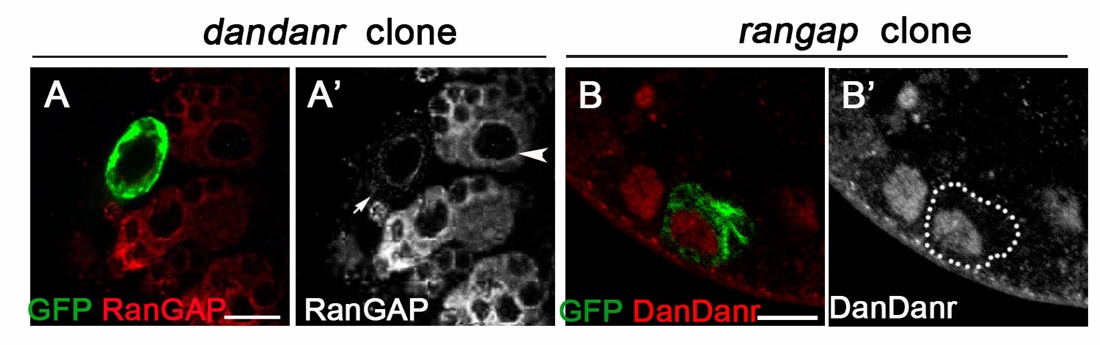


**Figure S6: Dan/Danr and RanGap relationship**

**(A - A')** *dan/danr* NB clone stained by RanGAP (red). GFP (green) labels the clone. Arrow indicates the NB in clone and arrowhead marks the counterpart of control. **(B - B')** *rangap* NB clones stained by DanDanr (red). GFP (green) labels the clone. Arrow indicates the NB in clone and arrowhead marks the counterpart of control. Scale bars: 10 μm.)
